# Fatal Errors

**Published:** 1881-04

**Authors:** Ad. Lippe

**Affiliations:** Phila.


### FATAL ERRORS.

BY AD. LIPPE, M.D., PHILA.

IT is a fatal error to quote Hahnemann as saying "*Local applications of the drug to the sound skin greatly facilitate the action of the remedy.*"

This false assertion is made in the introductory to vol. xviii of the *American Observer*, Jan. 12th, 1881. This Journal flourishes on the title page the homœopathic formula, *Similia Similibus Curantur*. The introduction alluded to surpasses any thing we have ever read; there are misrepresentations and utterly false quotations; there are assertions too absurd to be tolerated, all seasoned by unprecedented malice. The "Internationals" are told that "freedom of medical opinion and action" is not to be extended to them; they are accused of advocating opinions they never thought of, and to crown this masterwork of falsifications the *learned* editor exposes not only his malicious spirit, but also his utter ignorance of Hahnemann's writings. If the *learned* author of the introductory does not possess an Organon of the healing art, which we very much doubt, and if he can not find a copy among the habitual readers of this *Observer*, which we also doubt, he may probably be able to borrow Stratton's translation of the work from one or the other of the better read professors at Ann Arbor, who no doubt will be glad to help so *learned* an author as the writer of the introductory. And please ask for Stratton's translation and not for the mis-translation of Conrad Wesselhoeft, in which to find a shadow of Hahnemann's teachings would require a better microscope than has yet been invented. Now this *learned* author tells us that Hahnemann says "Local applications of the drug to the sound skin greatly facilitates the action of the drug," and that the wicked Internationals, who disturb the peace of the eclectic tribe, who want to annihilate homœopathy, have said "local applications are worse than useless."

In paragraph 194 of his "Organon," the master says, "under no circumstances should the homœopathically indicated and internally administered medicine be applied locally." And in § 197 the master declares that it is objectionable to administer in diseases showing themselves locally the same remedy externally as is administered internally. Notwithstanding the fact, that the local applications will the sooner remove the external symptoms; but that such local application is, *for that very reason*, to be rejected. For, as the internal disease still continues, the disappearance of the local symptoms does not constitute a radical cure; indeed, this radical cure becomes more precarious and in some instances impossible, after these local applications have removed the local symptoms. So says Hahnemann, and he is in this, as in other points, endorsed by the experience of every true homœopathician. It is the duty of the public teachers to teach their students to avoid this error. It is a fatal error, of which the author of the introductory is also guilty, to suppose that every preceptor who sends his student to college can demand from the professors *not* to contradict him, however ignorant *he* may be; what a state of affairs would that be? Again, it is a fatal error to suppose, as the *learned* author has it, that Hahnemann ever said, "In urgent and dangerous cases palliative measures are admissible and proper." Where did Hahnemann say any such thing?

The Internationals have a good right to express their convictions that just in the most urgent and dangerous cases palliatives are most pernicious, that they reject them, and are ready to warn the public against ignorant pretenders who resort to those palliatives and then boldly claim that Hahnemann approved of such miserable, humbugging, allopathic practice.

